# Minimally Invasive Repair of Aneurysmal Coronary-Pulmonary Artery Fistulas

**DOI:** 10.1016/j.atssr.2022.09.010

**Published:** 2022-09-26

**Authors:** Kazuki Noda, Naonori Kawamoto, Satoshi Kainuma, Ayumi Ikuta, Naoki Tadokoro, Takashi Kakuta, Tomoyuki Fujita, Satsuki Fukushima

**Affiliations:** 1 Department of Cardiovascular Surgery, National Cerebral and Cardiovascular Center, Suita, Osaka, Japan

## Abstract

The surgical indication for aneurysmal coronary-pulmonary artery fistula (CPAF) has not yet been established. We present 2 cases of aneurysmal CPAFs successfully treated with bilateral minithoracotomy. Case 1 involved a 40-year-old woman who presented with asymptomatic CPAFs and aneurysms measuring 25 mm in diameter. Considering her cosmetic concerns, CPAFs were surgically treated through minithoracotomy, with no major postoperative complications. In case 2, an 82-year-old woman with dyspnea was diagnosed with high-flow coronary-pulmonary artery shunts with aneurysms. Given her age and frailty, surgical closure through minithoracotomy was indicated for the symptoms of heart failure caused by significant left-to-right shunts.

A coronary-pulmonary artery fistula (CPAF) has been found in 15% to 20% of the population, whereas aneurysmal formation has been found in 19%.^1^^,^^2^ In the case of multiple fistulas complicated by large aneurysms, open surgical repair is usually preferred to endovascular treatment.^3^ However, few reports have mentioned a minimally invasive surgical approach in such cases.

## Case Reports

### Patient 1

**Figure 1:**
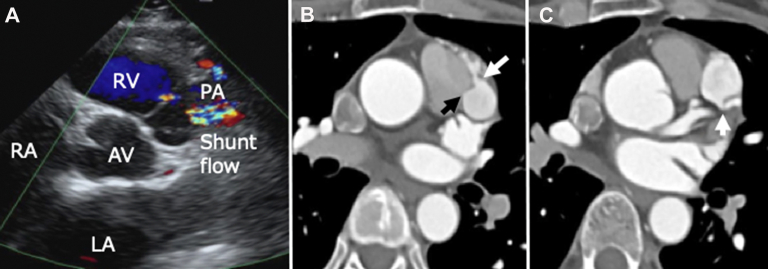
(A) The Doppler color image showed a continuous shunt flow to the main pulmonary artery (PA) on echocardiography. Enhanced computed tomography showed the aneurysm connecting to the (B) main pulmonary artery (black arrow) and branches of the right coronary artery (white arrow) and (C) left anterior descending coronary artery (arrow). (AV, aortic valve; LA, left atrium; RA, right atrium; RV, right ventricle.)

**Figure 2:**
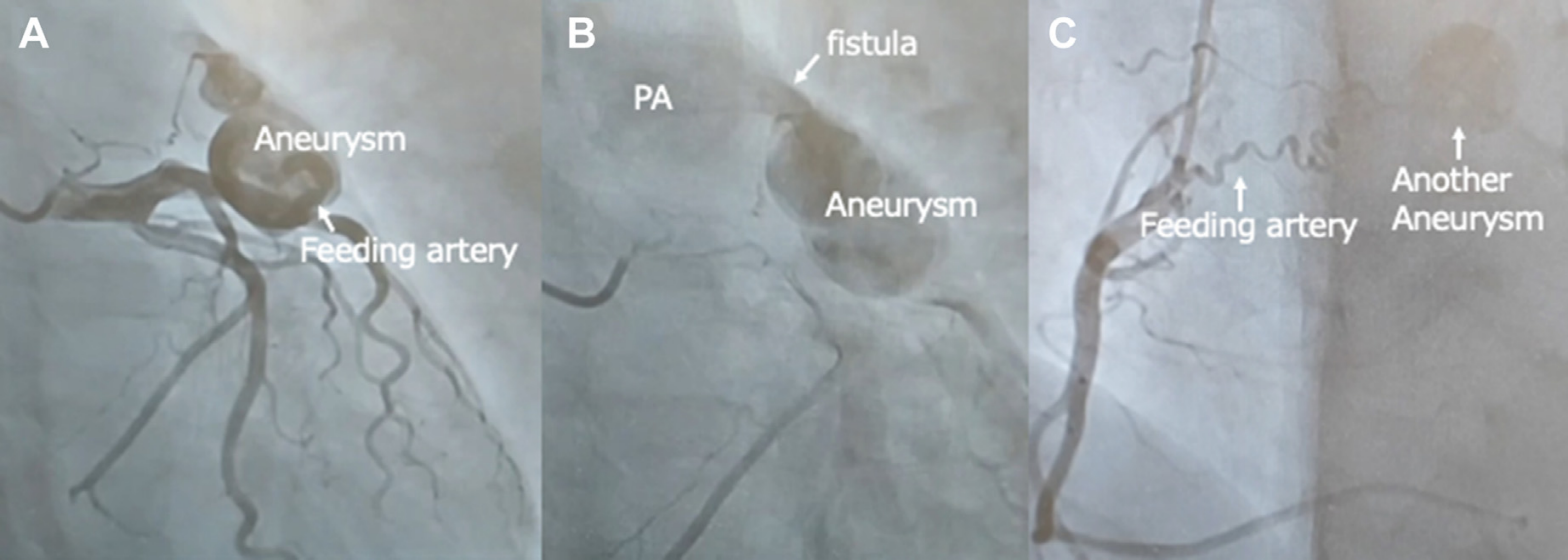
Left coronary angiography demonstrates (A) inflow tracts originating from the left anterior descending coronary artery into the aneurysm, (B) fistulizing into the pulmonary artery (PA) in the late phase. (C) Right coronary angiography shows the anomalous artery from right coronary artery feeding into another aneurysm.

A 40-year-old woman presented with an inverted T wave from V_2_ to V_4_ on the electrocardiogram and a protrusion of the left third bow on the chest radiograph at her annual health examination. A continuous shunt flow from the left coronary artery to the pulmonary artery was revealed by echocardiography (Figure 1A). Computed tomography (CT) angiography revealed multiple aneurysms with a maximum diameter of 25 mm and several inflow tracts that originated from the proximal left anterior descending coronary artery (LAD) and the proximal right coronary artery (RCA) and fistulized to the main pulmonary artery (Figures 1B, 1C). Left coronary angiography showed inflow tracts that originated from the LAD (Figure 2A), fistulizing into the pulmonary artery in the late phase (Figure 2B). Right coronary angiography showed the anomalous artery from the RCA feeding into another aneurysm (Figure 2C). The ratio of pulmonary-to-systemic blood flow (Qp/Qs) was 1.06 on the basis of right-sided heart catheterization.

Given the patient’s large aneurysms and multiple feeding arteries, we decided to perform surgical closures through bilateral minithoracotomy, considering her desire for cosmetic aspects.

During the bilateral minithoracotomy approach, the patient was intubated with a double-lumen endotracheal tube. Before draping, the right jugular vein was cannulated for cardiopulmonary bypass (CPB).

Based on the preoperative multidetector CT scan, which revealed an accurate positional relationship between the aneurysm and the ribs, a bilateral minithoracotomy approach was used with a 4-cm incision made through the right and left fourth intercostal spaces. A soft tissue retractor was used to limit rib spreading. CPB was initiated through the right femoral artery, followed by vacuum-assisted venous drainage through the right femoral vein and right internal jugular vein. A root cannula was inserted into the ascending aorta for antegrade cardioplegia, venting, and pressure monitoring. Cardiac arrest was induced by direct cross-clamping of the ascending aorta and obtained with antegrade cardioplegia. Through the left fourth intercostal space, aneurysms were well observed directly. After an aneurysm was opened, the inflow tracts from the LAD and RCA as well as the drainage vessels to the pulmonary artery were recognized and directly ligated.

**Figure 3:**
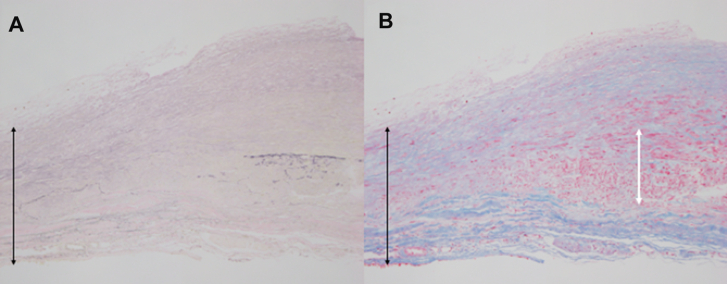
(A) The ruptures and disappearance of the internal elastic membrane (black arrow) stained with elastica van Gieson. (B) Hematoxylin and eosin staining revealed that some parts of the tunica media retained the smooth muscle layer (white arrow); however, the layer replaced the fibrosis tissues in the part of aneurysms (black arrow).

Postoperative enhanced CT revealed no residual fistula, and she was discharged home without complications on postoperative day 9. Pathologic examination revealed that the internal elastic membrane had ruptured (Figure 3A), and the tunica media of the aneurysms was thin, with atrophy of the vascular smooth muscle (Figure 3B).

### Patient 2

An 82-year-old woman complained of progressive dyspnea. Enhanced CT confirmed the presence of multiple coronary artery aneurysms with a maximum diameter of 25 mm. In addition, feeding arteries were arising from the proximal LAD and RCA and draining to the main pulmonary artery. The Qp/Qs was 1.5 on blood gas analysis. Given the patient’s age and frailty, CPAF closure was indicated through a bilateral minithoracotomy approach because of her heart failure symptoms and significant left-to-right shunts.

Cardiac arrest was achieved in the same way as with the first patient. The aneurysm was precisely exposed through the left fourth minithoracotomy. After the aneurysm was opened, the inflow from the LAD and RCA and the drainage vessels to the pulmonary artery were directly ligated. Postoperative enhanced CT showed no residual fistula; 1 year after discharge, she is ambulatory with no symptoms of heart failure.

## Comment

The appropriate indication for treating CPAF has remained controversial. Several reports found that high Qp/Qs (1.3-1.5) and the presence of large aneurysms are indications for closure.^4^ Previous reports have presented that CPAFs with aneurysms >30 mm in diameter are at risk of rupture.^5^ However, there have been some reports of rupture in patients with small aneurysms of 10 to 15 mm.^6^ On pathologic examination, rupture of the internal elastic membrane was found in our first case, and the tunica media became thin with smooth muscle atrophy, indicating that the patient was at high risk of free wall rupture after cardiac tamponade. Therefore, CPAFs with aneurysms of any size should be considered a surgical indication.

According to Mangukia,^7^ open surgical closure is preferable in patients with large fistulas with multiple communications and large aneurysms. Recently, with the advancement of surgical techniques and devices, the minimally invasive cardiac approach has become increasingly popular because of better cosmetic results, less pain, and faster recovery. Song and colleagues^8^ reported that a minimally invasive surgical procedure for CPAF that included a minithoracotomy but did not include CPB had an excellent outcome. However, reports of a minithoracotomy approach with CPB for patients with CPAF and aneurysm are uncommon. To prevent residual shunt and recurrent aneurysms, the aneurysms must be opened under CPB, and multiple feeding arteries must be securely ligated. To provide accurate location of the CPAF and aneurysms, multidetector CT with 3-dimensional reconstruction is necessary, which successfully guides the minithoracotomy approach.

In conclusion, for multiple CPAFs complicated by large coronary artery aneurysms, surgical closure with a bilateral minithoracotomy is safe and feasible, and it can be an alternative to a median sternotomy approach for surgical treatment.
